# Laboratory Surveillance of Dengue in Rio Grande do Sul, Brazil, from 2007 to 2013

**DOI:** 10.1371/journal.pone.0104394

**Published:** 2014-08-12

**Authors:** Gabriela Luchiari Tumioto, Tatiana Schäffer Gregianini, Bibiana Paula Dambros, Beatriz Carneiro Cestari, Zenaida Marion Alves Nunes, Ana Beatriz Gorini Veiga

**Affiliations:** 1 Laboratory of Molecular Biology, Universidade Federal de Ciências da Saúde de Porto Alegre (UFCSPA), Porto Alegre, RS, Brazil; 2 Graduate Program in Pathology, Universidade Federal de Ciências da Saúde de Porto Alegre (UFCSPA), Porto Alegre, RS, Brazil; 3 Fundação Estadual de Produção e Pesquisa em Saúde – Instituto de Pesquisas Biológicas – Laboratório Central de Saúde Pública do Rio Grande do Sul (FEPPS IPB-LACEN/RS), Porto Alegre, RS, Brazil; Public Health England, United Kingdom

## Abstract

**Background:**

According to official records, dengue was introduced in Brazil in the 80's; since then several epidemics have occurred. Meanwhile, in Rio Grande do Sul (RS, Southern Brazil) the first autochthonous case occurred only in 2007.

**Methodology and Principal Findings:**

In this study we report laboratory surveillance of dengue cases and seasonality of positive cases, describe serotypes and characterize the epidemiological pattern of dengue in RS from 2007 to 2013. A total of 9,779 serum samples from patients with suspected dengue fever were collected and submitted to molecular and/or serological analyses for dengue virus identification and serotyping, based on viral isolation, NS1 antigen detection and qRT-PCR, or Dengue IgM capture ELISA and MAC-ELISA. The first autochthonous dengue case in RS was confirmed in 2007 (DENV-3). While in 2008 and 2009 only imported cases were registered, autochthonous infection waves have been occurring since 2010. The highest number of dengue infections occurred in 2010, with DENV-1 and DENV-2 outbreaks in Northwestern RS. In 2011, another DENV-1 and DENV-2 outbreak occurred in the Northwestern region; moreover, DENV-4 was detected in travelers. In 2012, DENV-1 and DENV-4 co-circulated. DENV-2 circulation was only detected again in 2013, in high frequency (56.7%), co-circulating with DENV-4 (35%). Most infections occur in adults during summer. Differences in prevalence between genders were observed in 2007 (60% females), 2008 (60.8% males) and 2009 (77.5% males).

**Conclusions:**

According to results of dengue surveillance, there was an increase in the number of dengue cases in RS and of cities infested with *Aedes aegypti*, possibly as a consequence of introduction of new serotypes and the difficulty of health programs to control the vector.

## Introduction

Dengue fever (DF) is the most important arthropod-borne viral disease and is considered a serious public health problem [1], affecting between 50-100 million people/year worldwide, causing 250,000 cases of Dengue Hemorrhagic Fever (DHF). Moreover, 2.5 billion people live in dengue-endemic countries, including Brazil [2].

The clinical response to infection by dengue virus (DENV) causes a spectrum of disease symptoms [3], [4], from mild DF to severe DHF and Dengue Shock Syndrome (DSS). DENV is transmitted by mosquitoes of the genus *Aedes* and consists of four closely related but antigenically distinct serotypes (DENV-1, DENV-2, DENV-3 and DENV-4) [4]. All four serotypes can be distinguished by serological and molecular methods [5], [6].

In Brazil, there are reports of dengue epidemics in 1916 and 1923, in the States of São Paulo and Rio de Janeiro (Southeast Brazil), respectively. However, the first laboratory-confirmed DENV case was reported in the State of Roraima (Northern Brazil) only in 1981–1982, with the introduction of DENV-1 and DENV-4, probably an extension of the DENV epidemics waves that had occurred in Central America and in northern parts of South America [1].

In 1986–1987, DENV-1 caused an outbreak in Rio de Janeiro and then spread to other Brazilian states; in 1990, DENV-2 was also introduced in Rio de Janeiro [1], [7]. During the 90's the vector *Ae. aegypti* spread throughout the national territory and led to dissemination of DENV-1 and DENV-2 to 20 of the 27 Brazilian States [7].

The first dengue cases caused by DENV-3 were reported in 2000 in Rio de Janeiro, and in 2001 in Roraima. Subsequently, it was identified in the entire country, except in the Southern states of Santa Catarina and Rio Grande do Sul (RS) [1].

Since its first introduction in the country in the late 80's, DENV-4 was identified again in Brazil only in 2008, in the city of Manaus, State of Amazonas (Northern Brazil) [8]. In the following years, DENV-4 spread nationwide and started to circulate together with the other serotypes in different regions of the country.

Today, all four serotypes circulate in Brazil, and dengue is a mandatory notifiable disease. Historically, the State of RS has registered dengue circulation since 1996, with imported cases of people coming from other areas [9]. Subsequent autochthonous cases were detected from January 2007 to May 2013, with dengue outbreaks caused by DENV-1, DENV-2 and DENV-4 [10]-[13].

We report here the results of Dengue laboratory surveillance since the first autochthonous case identified in January 2007 until May 2013 in RS. This is the first time-series report on dengue epidemiology in RS, Brazil.

## Materials and Methods

### Ethics Statement

This study has been approved by the Ethics Committee of IPB-LACEN/RS (Ethics Statement n. 371.278) and of UFCSPA (Ethics Statement n. 332.274). Both Ethics Committees waived the need for written informed consent from the donors because samples were routinely received at IPB-LACEN/RS for laboratory surveillance of dengue and not for diagnosis of the patients. Dengue notification has become compulsory in Brazil and all information obtained is for epidemiological surveillance and research purposes.

### Patients and Samples

This study included human serum samples sent to the Central Laboratory of Public Health (IPB-LACEN/RS) for dengue surveillance diagnosis, including all patients with clinically suspected DF in RS. Clinical DF was based on the following symptoms: sudden onset of fever, headache, retro-orbital pain, body aches, nausea and vomiting, joint pains, weakness, and rash [4]. For each patient, the *Dengue Notification Form* – containing information such as demographic characteristics, date of notification, timing of sampling, symptoms, travel history, results of laboratory tests – was filled out at time of sample collection. Samples were analyzed using commercially available tests (NS1 antigen detection and Dengue IgM capture ELISA), and in-house IgM antibody capture ELISA (MAC-ELISA) as well as real time reverse transcription-polymerase chain (qRT-PCR) and viral isolation, depending on the days of onset of symptoms, as described below.

Dengue-positive serum samples that had been previously analyzed and serotyped in our laboratory were used as positive controls in qRT-PCR and MAC-ELISA assays.

### Dengue Assays

The Brazilian National Dengue Program states that samples collected within five days of the onset of symptoms must be analyzed either by the NS1 antigen detection assay followed by qRT-PCR for confirmation (when NS1 antigen is positive), or directly submitted to viral isolation for confirmation and DENV serotyping. On the other hand, samples that are collected after six or more days of the onset of symptoms must be analyzed based on the serological method using the Dengue IgM Capture enzyme-linked immunosorbent assay (ELISA); positive samples are then confirmed by MAC-ELISA [14]. Accordingly, only results obtained either with viral isolation, qRT-PCR and/or MAC-ELISA are valid for confirming dengue diagnosis.

The laboratory surveillance of all suspected dengue cases in RS is performed in IPB-LACEN/RS. From 2007 to 2010, IgM-based assays were performed at IPB-LACEN/RS and at the Adolfo Lutz Institute (IAL); serotyping based on viral isolation [13] was done at IAL. Since 2011, IPB-LACEN/RS has been in charge of all analyses, including DENV detection and serotyping, as well as serological tests, according to the methodologies described below.

#### NS1 Antigen Detection

NS1 antigen detection was performed with the Platelia™ Dengue NS1 Ag kit (BIO-RAD Laboratories, France), according to the manufacturer's instructions.

#### DENV Serotyping by qRT-PCR

Dengue virus serotyping was based on qRT-PCR as described by Johnson *et al*. (2005) [15]. Total viral RNA was extracted from 200 µL of NS1-reagent serum specimens using Pure Link Viral RNA/DNA Mini Kit (Invitrogen-Life Technologies, USA). In singleplex reaction mixtures, 7 µL of viral RNA was combined with 25 pmol of each primer and 4.5 pmol of the dye-labeled probe in a 25 µL reaction volume, using the SuperScriptIII Platinum One-Step Quantitative RT-PCR System (Invitrogen-Life Technologies, USA), according to the manufacturer's instructions. Reaction conditions were 20 min at 50°C; 2 min at 95°C, 45 cycles of 95°C for 15 s and 60°C for 30 s, in a 7500 Real-Time PCR System (Applied Biosystems-Life Technologies, USA).

#### Antibody Tests (IgM)

For serological tests, the Dengue IgM capture ELISA kit (Panbio-Inverness Medical Innovations, Australia) was used, according to manufacturer's instructions. Positive samples were confirmed using MAC-ELISA according to Kuno *et al*. [16]. Antigens for this test were provided by Evandro Chagas Institute, State of Pará.

### Statistical Analyses

Descriptive statistics for the study population characteristics and laboratory findings were performed using SPSS Inc. (Chicago, IL, Version 17.0). *X^2^* test was applied for comparisons among proportions (standardized adjusted residuals ≥2 was used when the number of the cases observed was greater than the expected) and *p*<0.05 was considered significant.

## Results

A total of 9,779 samples collected for suspected dengue fever were analyzed between January 2007 and May 2013, of which 2,046 (21%) were positive for dengue based on any of the tests used. From 2007 to 2010, samples collected within five days of the onset of symptoms were analyzed by viral isolation, confirming 25 cases. From 2011 to 2013, all samples were analyzed by the NS1 antigen detection assay, and positive samples (175 cases) were confirmed by qRT-PCR. Samples collected after the fifth day of the onset of symptoms were analyzed by the Dengue-IgM capture ELISA, and positive samples were then submitted to analysis by MAC-ELISA, confirming 1,846 cases from 2007 to 2013 (Table 1).

**Table 1:** Dengue cases reported in the State of Rio Grande do Sul, Brazil, from January 2007 to May 2013.

Year	Total	Positive	Positive/Total (%)	Methodology		
				Viral isolation	qRT-PCR	MAC-ELISA
2007	1,529	238	15.6	2	N.A.	236
2008	917	97	10.6	0	N.A.	97
2009	297	40	13.5	0	N.A.	40
2010	2,345	900	38.4	23	N.A.	877
2011	1,772	276	15.6	N.A.	55	221
2012	681	119	17.5	N.A.	23	96
2013	2,238	376	16.8	N.A.	97	279
Total	9,779	2,046	20.9	25	175	1,846

N.A.: not analyzed.

**Figure 1:**
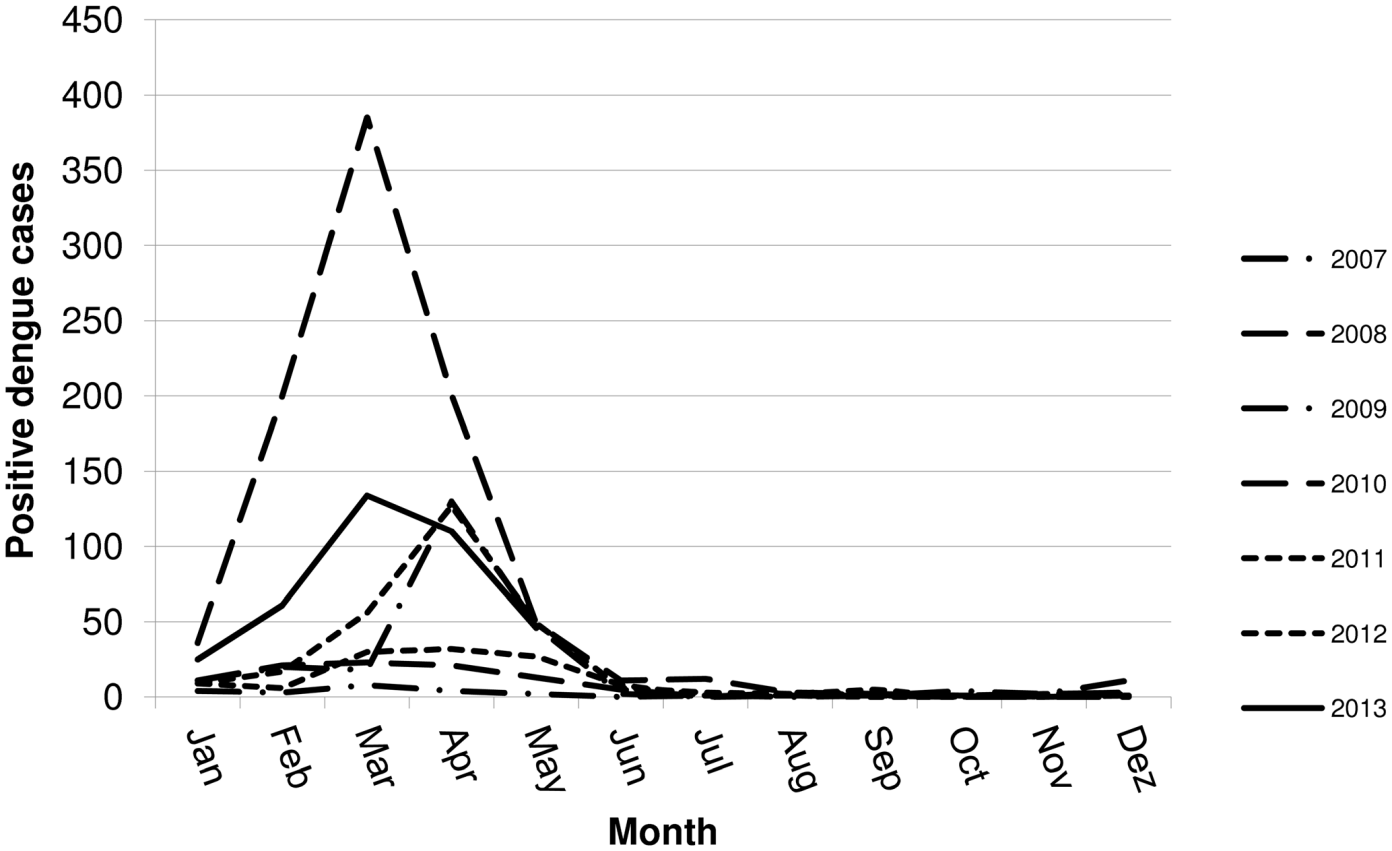
Seasonality of positive dengue cases in the State of Rio Grande do Sul, Southern Brazil, according to months (2007–2013).

In the state of Rio Grande do Sul dengue infection cases occurred during summer, displaying a peak in March and April, while few cases are reported from July to November (winter and spring) and these are often imported cases; the number of cases starts to increase again in January and February. The monthly distribution of dengue-positive cases along the years (2007-2013), in RS, is shown in Figure 1.

**Figure 2:**
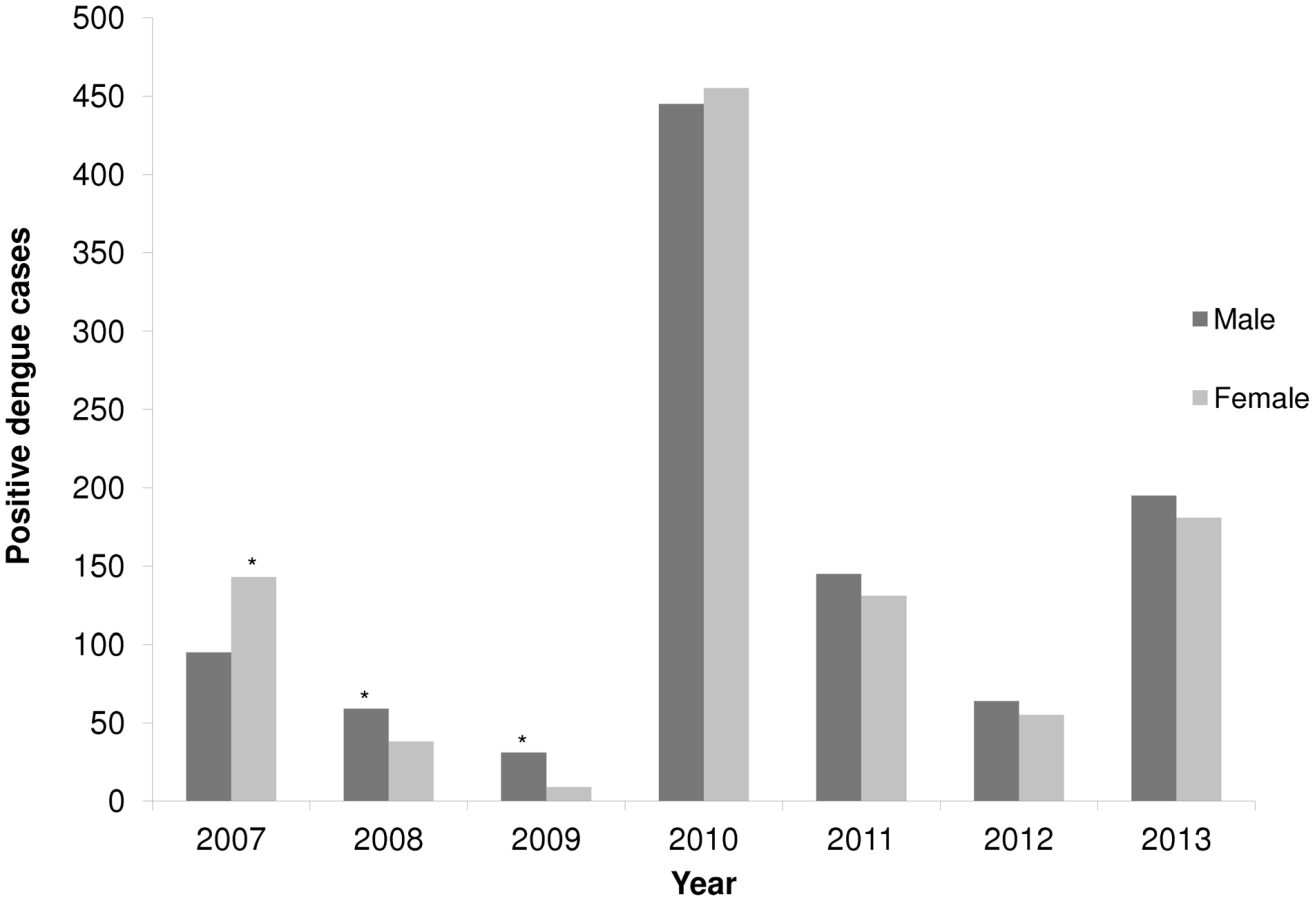
Frequency distribution of positive dengue cases in the State of Rio Grande do Sul, according to gender (2007–2013).

In 2007, a total of 1,529 dengue suspected cases were notified and 15.6% (238) were positive based on MAC-ELISA and viral isolation (Table 1). The frequency was similar in the age groups analyzed (Table 2), and higher in female than in male (143 and 95, respectively, Figure 2). The DENV-3 serotype was detected in that year.

**Table 2:** Dengue cases in the State of Rio Grande do Sul according to age (2007–2013)^a^.

Year	2007	2008	2009	2010	2011	2012	2013	Total
Age (years)	n (%)	n (%)	n (%)	n (%)	n (%)	n (%)	n (%)	n
<1–10	8(3.4)	7(7.2)^b^	1(2.5)	39(4.3)^b^	4(1.4)	1(0.8)	10(2.7)	70
11–20	0	6(6.2)	3(7.5)	118(13.1)^b^	30(10.9)	10(8.4)	50(13.3)	217
21–30	36(15.1)	22(22.7)	7(17.5)	126(14)	51(18.5)	35(29.4)^b^	80(21.3)^b^	357
31–40	40(16.8)	21(21.6)	11(27.5)	141(15.7)	58(21)	17(14.3)	78(20.7)	366
41–50	51(21.4)	21(21.6)	6(15)	175(19.4)	57(20.7)	23(19.3)	69(18.4)	402
51–60	35(14.7)	7(7.2)	7(17.5)	110(12.2)	43(15.6)	18(15.1)	58(15.4)	278
>60	21(8.8)	6(6.2)	0	138(15.3)^b^	31(11.2)	15(12.6)	31(8.2)	242
N.I.^c^	47(19.7)	7(7.2)	5(12.5)	53(5.9)	2(0.7)	0	0	114
Total	238	97	40	900	276	119	376	2,046

a Number of positive cases and percentages are ratio among positive cases by total samples tested by year.

b Standardized adjusted residuals ≥2 according to data of test χ^2^ = 294.49, *p*<0.001.

c N.I.: not informed (data was not informed in the *Dengue Notification Form*).

During 2008 only imported cases were reported and computed. Of the 917 notifications, 10.6% were dengue positive, with higher prevalence in the age group under 10 years old; the frequency of infection was higher among males (60.8%). There were only imported cases in that year; the few samples had been collected with more than five days after the onset of symptoms, thus serotyping was not possible.

In 2009, only imported dengue cases were computed as well, with fewer notifications (297) than in 2008. Positivity was 13.5%, the most affected gender was male (77.5%), and no difference among age groups was found. There is no information about the serotypes circulating in 2009 because collected samples were inappropriate to viral isolation.

In 2010, a total of 2,345 suspected cases of dengue were reported in RS, among which 41.7% were autochthonous (375/900). This was the year with the highest number of dengue infections (38.4%). Viral isolation confirmed DENV-1 outbreak in Santa Rosa (14.5%) and DENV-2 in Ijuí (39%) and Santo Ângelo (9.4%), cities located in the Northwest region of RS (near the border with the state of Santa Catarina and with Argentina). The most affected age groups were young people aged up to 20 years and above 60 years old. A total of 397 patients reported travel within the RS and other regions.

DENV-1 was detected in 92.7% of the cases serotyped in 2011. Another outbreak occurred in Santa Rosa with concomitant circulation of DENV-1 (47.8%) and DENV-2 (with low frequency). Moreover, in that year DENV-4 was first detected in RS in three (5.5%) people who arrived from Northern Brazil.

In 2012, an outbreak occurred again in Santa Rosa with concomitant circulation of DENV-1 and DENV-4; the latter was the most (56.5%) common serotype in RS in that year. The age group 21–30 years old was the most affected. DENV-2 was not detected in 2012 in RS. On the other hand, DENV-2 circulation was detected again in 2013 in high frequency (56.7%), as well as DENV-4 (35%). A large number of patients (2,238) were investigated in 2013 and 21-30 years old were the most affected group. DENV-1 and DENV-4 were detected in 13% (49/376) of the dengue cases in Santa Rosa, and DENV-1, DENV-2 or DENV-4 were detected in 50.8% (191/376) of patients from Porto Alegre (capital city of RS, Eastern region).

Table 3 resumes the DENV serotypes circulating in RS in each year, from January 2007 to May 2013.

**Table 3:** Dengue cases in the State of Rio Grande do Sul according to serotypes (2007–2013).

Year	Serotypes(%)^a^			
	DENV-1	DENV-2	DENV-3	DENV-4
2007	–	–	2(100)	–
2008	–	–	–	–
2009	–	–	–	–
2010	4(15.4)	22(84.6)	–	–
2011	51(92.7)	1(1.8)	–	3(5.5)
2012	10(43.5)	–	–	13(56.5)
2013	8(8.3)	55(56.7)	–	34(35)

a Percentages are ratio among dengue serotypes by positive samples.

## Discussion

According to official records, dengue virus was introduced in Brazil in the 80's, and since then, all serotypes have been circulating in different regions of the country. In RS, Southern-most State of Brazil, the first imported dengue cases were notified in 1996, and the first autochthonous cases were confirmed in 2007 in cities located in Northwestern RS (Giruá, Horizontina, Tucurundi, Três de Maio) and Northern RS (Erechim) [17]. Since, autochthonous dengue transmission has occurred in RS, as it has been well documented in other regions where *Aedes aegypti* mosquitoes are present.

In the years that RS registered autochthonous cases, difference between male and female was observed only in 2007, when the prevalence was more frequent in females (60%); on the other hand, in 2008 and 2009, when all cases were imported, dengue was more frequent in males (60.8% and 77.5%, respectively), probably associated to more mobility of adult men.

In the majority of Brazilian States, DENV-3 was prevalent from 2002 to 2006, being displaced by DENV-2 from 2007 to 2009 [18]. On the other hand, during the first years of dengue notification in RS, the circulating serotypes followed a dynamics different from what was observed in other Brazilian regions. Accordingly, DENV-3 was identified in autochthonous samples in RS in 2007, then only imported cases occurred in 2008 and 2009 in the State. In 2010, DENV-2 was the most common serotype in RS, while in Brazil there was a higher rate of DENV-1 [10].

Between 2011 and 2013, the dynamics of circulating dengue serotypes were similar in RS and in the rest of the country (Ministério da Saúde/Secretaria de Vigilância em Saúde MS/SVS, unpublished data). In 2011 and 2012, DENV-2 was gradually displaced by DENV-4; notably, while DENV-4 was identified in only 5.5% of the cases in 2011, in 2013 it accounted for 35% of the cases, spreading through eight cities in RS. Regarding DENV-2, after a low circulation in 2011 and a silent circulation in 2012, this serotype reemerged in 2013 in 56.7% of the cases. Inversely, DENV-1 decreased from 92.7% in 2011 to 8.3% in 2013 in RS.

A previous study based on phylogenetic analysis of DENV-4 that circulated in RS and in the State of São Paulo in 2011, showed that the Brazilian strains are closely related to strains that have been circulating since 1981, when DENV-4 was first introduced in South America, suggesting that the virus has gone through recent evolution for at least 4 to 6 years [13]. DENV-4 may have penetrated the Brazilian population earlier than 2010, causing milder disease; therefore, and also due to a higher prevalence of DENV-1 and DENV-2, the virus could have been present but not detected. The reemergence of DENV-4 evidences that the replacement of a dominant circulating genotype is associated with the rising of a previously rare lineage [13].

The highest number of notifications and many autochthonous cases occurred in 2010, the year with the highest number of positive dengue infections (38.4%), compared to other years when the frequency did not exceed 17.5%. In 2011, 276 infections occurred and 18% were in Porto Alegre. Even though there were fewer notifications in 2012, dengue infections increased by 23.5% in Porto Alegre. Thereafter, the number of cities with autochthonous dengue cases increased, from five cities in 2007 to 53 in 2013.

Even though the number of dengue cases in RS increased in the last years, it was proportionally lower than in other regions of Brazil [19]. This condition may be associated with the climate of the region, with rigorous winters that are not suitable for mosquitoes reproduction, different from the rest of Brazil where temperatures are high year-around [3].

Control of DENV spread relies primarily on reduction of vector populations, which is usually done by elimination of still water sources and use of insecticides. Despite the effort of the State Program for Surveillance of *Ae. aegypti*, detection of the vector increased in RS [20], confirming the persistence of infested cities and the emergence of new occurrences. In 2007 there were 52 cities infested with *Ae. aegypti*, while in May 2013 this number has risen to 116 (496 cities), making of RS an endemic region.

Molecular studies are also fundamental to understand viral evolution, viral dynamics and its interaction with hosts, and to predict the severity of the disease. DENV phylogenetics and phylodynamics in RS should be taken into account in future studies. This approach will contribute to improve the quality of surveillance data, to predict circulation of different dengue serotypes and genotypes as well as to evaluate the impact of preventive measures such as mosquito control or a future vaccine.

Finally, considering that dengue was recently introduced in RS, the control of further outbreaks requires a network composed of well-trained and skilled health professionals, entomologists, environment surveillance agents, laboratory technicians and molecular biologists, as well as social education and governmental support.

## Supporting Information

Table S1 Data of reported dengue cases in 2007.
(XLS)

Table S2 Data of reported dengue cases in 2008.
(XLS)

Table S3 Data of reported dengue cases in 2009.
(XLS)

Table S4 Data of reported dengue cases in 2010.
(XLS)

Table S5 Data of reported dengue cases in 2011.
(XLS)

Table S6 Data of reported dengue cases in 2012.
(XLS)

Table S7 Data of reported dengue cases in 2013.
(XLS)
